# Type B insulin resistance syndrome induced by anti‐PD‐1 therapy

**DOI:** 10.1111/1753-0407.13603

**Published:** 2024-09-12

**Authors:** Xiaomin Shi, Mengyu He, Li Ni, Zhijuan Dai, Mengte Shi, Yingying Zhou, Huabing Zhang, Ming Li, Chaoming Wu

**Affiliations:** ^1^ Department of Endocrinology The Second Affiliated Hospital and Yuying Children's Hospital of Wenzhou Medical University Zhejiang China; ^2^ Department of Endocrinology, Key Laboratory of Endocrinology of National Health Commission, Peking Union Medical College Hospital Chinese Academy of Medical Sciences & Peking Union Medical College Beijing China; ^3^ Department of Laboratory Medicine The Second Affiliated Hospital and Yuying Children's Hospital of Wenzhou Medical University Zhejiang China

Dear Editor,

A 59‐year‐old man was diagnosed with Hodgkin lymphoma in May 2020 and began treatment with sintilimab in August 2020. The patient had a normal blood glucose test before receiving treatment with sintilimab, with no family history of diabetes. In September 2020, the patient developed diabetic ketoacidosis after receiving three cycles of sintilimab. Glycated hemoglobin A1c (HbA1c) was 7.0%, and C‐peptide level was undetectable. Diabetes‐related antibodies, including glutamic acid decarboxylase antibody, insulinoma‐associated protein 2 antibodies, and insulin autoantibodies, were all negative. Based on these findings, he was diagnosed with fulminant type 1 diabetes caused by anti‐programmed cell death‐1 (anti‐PD‐1) therapy. Additionally, he was also diagnosed with destructive thyroiditis caused by anti‐PD‐1 therapy at the same time. After discharge, he received insulin therapy, and his glucose level fluctuated between 4 and 20 mmol/L. The treatment regimen for Hodgkin's lymphoma was modified, and sintilimab treatment was stopped. Two months later, the Hodgkin's lymphoma was resolved.

In November 2021, the patient was admitted to the endocrinology department due to significant weight loss. In the 3 months leading up to his admission, he had lost about 15 kg in weight, with a poor glucose control (often >33.3 mmol/L). He did not report any symptoms of nausea or vomiting. On physical examination, he weighed 47 kg and had a body mass index (BMI) of 16.65 kg/m^2^. On admission, his plasma glucose level was 29.9 mmol/L, and β‐hydroxybutyric level was 0.3 mmol/L. Of note, his serum C‐peptide was <0.05 ng/mL, while his serum insulin level was >300 mU/L, and his HbA1c level was 10.2%. He was commenced on intravenous regular insulin therapy, and the insulin dose was gradually increased. However, despite continuous intravenous infusion of up to 3200 U of regular insulin daily, his blood glucose was still above 15 mmol/L. Other examinations showed that serum triglyceride was 0.89 mmol/L, adiponectin was 25.15 μg/mL, and insulin‐like growth factor‐1 was <25 ng/mL. Diabetes‐related antibodies were all negative as before.

We measured his serum insulin receptor antibody by using enzyme linked immunosorbent assay, which was developed in Key Laboratory of Endocrinology of National Health Commission, Peking Union Medical College Hospital. This patient had a positive result on the insulin receptor antibody test. He was therefore diagnosed with type B insulin resistance syndrome (TBIRS). During this admission, he was also diagnosed with Sjogren's syndrome and hemolytic anemia. We commenced him on an immunosuppressive regimen, combining iv rituximab (0.5 g), cyclophosphamide (0.4 g), and glucocorticoids. Glucocorticoids were administered as methylprednisolone 500 mg intravenously for 3 days, followed by a maintenance dose of 50 mg po daily, with a prolonged taper. Hydroxychloroquine was administered in a dose of 0.2 mg po daily continuously. After discharge, he received intensive insulin therapy. The patient's blood glucose control showed gradual improvement, with a total daily insulin dose of approximately 72 U. At week 65, his fasting blood insulin concentration was 23 mU/L (Table 1).

**TABLE 1:** Laboratory findings and treatment.

Week	0	10	17	25	39	47	65
Variable							
Fasting glucose (mmol/L)	18.2	19.5	17.2	8.9	5.3	8.0	5.0
Insulin (mU/L)	>300	>300	>300	>300	61.8	78.6	23
HbA1c (%)	10.2	12.4	17.2	14.9	10.4	10.2	8.8
CD19 (cells/μL)	704	1.1	0	3.2	13.6	14.1	58.8
ANA 1:1000	+		−		−		−
Anti‐SSA antibody	+		+		−		−
Anti‐SSB antibody	+		+		−		−
Treatment							
Rituximab	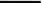						
Cyclophosphamide	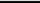						
Methylprednisolone	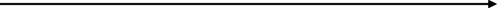						
Hydroxychloroquine	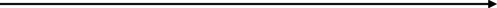						

Abbreviations: ANA, antinuclear antibody; Anti‐SSA antibody, anti‐Ro antibody; Anti‐SSB antibody, anti‐La antibody; CD19, CD‐19 B lymphocyte; HbA1c, glycated hemoglobin A1c.

We report a patient who developed fulminant type 1 diabetes 2 months after initiating treatment with sintilimab. Additionally, 1 year later, the patient presented with severe insulin resistance and was diagnosed with TBIRS. With the increasing use of immune checkpoint inhibitors, clinicians have started to focus on various immune‐related adverse effects (irAEs).^1^ However, there is currently no literatures reporting on anti‐PD‐1 therapy causing TBIRS. TBIRS is an intractable condition with a poor prognosis. Therefore, it should be considered as one of the severe irAEs, and further attention and investigation are warranted.

## AUTHOR CONTRIBUTIONS

Xiaomin Shi, Mengyu He, Li Ni, Zhijuan Dai, Mengte Shi, and Yingying Zhou performed the literature search, made the clinical diagnoses, performed the treatments, performed patient follow‐up, and wrote the manuscript. Huabing Zhang, Ming Li, and Chaoming Wu contributed to the study design, participated and supervised in the clinical diagnoses, and revised the manuscript. All authors were responsible for the interpretation of the data, revision, and final approval of the manuscript. Chaoming Wu is the guarantor of this work and, as such, had full access to all the data in the study and takes responsibility for the integrity of the data and the accuracy of the data analysis.

## CONFLICT OF INTEREST STATEMENT

This paper is our original unpublished work, and it has not been submitted to any other journal for reviews. We declare that the research was conducted in the absence of any commercial or financial relationships that could be construed as a potential conflict of interest.
